# Antimicrobial Susceptibility in Chicken-Associated Commensal Bacteria and Assembly-Linked Resistance-Gene Profiling of *Escherichia coli* from Northern Hungary

**DOI:** 10.3390/antibiotics15101002

**Published:** 2026-10-09

**Authors:** Ádám Kerek, Gergely Álmos Tornyos, Edit Szarka, Levente Hunor Husz, Máté Hetyésy, László Kovács, Ákos Jerzsele

**Affiliations:** 1 Department of Pharmacology and Toxicology, University of Veterinary Medicine Budapest, H-1078 Budapest, Hungary; tornyos.gergely.almos@student.univet.hu (G.Á.T.); szarka.edit@student.univet.hu (E.S.); husz.levente.hunor@student.univet.hu (L.H.H.); hetyesy.mate@student.univet.hu (M.H.);; 2 National Laboratory of Infectious Animal Diseases, Antimicrobial Resistance, Veterinary Public Health and Food Chain Safety, University of Veterinary Medicine Budapest, H-1078 Budapest, Hungary; kovacs.laszlo@univet.hu; 3 Department of Animal Hygiene, Herd Health and Mobile Clinic, University of Veterinary Medicine Budapest, H-1078 Budapest, Hungary

**Keywords:** antimicrobial resistance, laying hens, *Escherichia coli*, *Enterococcus*, *Staphylococcus*, minimum inhibitory concentration, antimicrobial resistance genes, *bla_CTX-M_*, Northern Hungary

## Abstract

**Background/Objectives**: Regional antimicrobial-resistance surveillance can be strengthened by linking phenotypic susceptibility data with genomic evidence. We characterized chicken-associated commensal bacteria from Northern Hungary and examined resistance-gene evidence in available *Escherichia coli* assemblies. **Methods**: Broth-microdilution minimum inhibitory concentration (MIC) data were available for 166 bacterial isolates collected in 2022–2023. Among the 69 *E. coli* isolates in this collection, 24 had corresponding draft whole-genome assemblies, defined here as assembled contig sets that had not been resolved into closed genomes; these assemblies were linked to the MIC records of the same isolates. Analyses included MIC distributions, descriptive correlations, principal component analysis, assembly-quality assessment, resistance-gene profiling, and sequence-context analysis. **Results**: The collection comprised 26 *Staphylococcus* spp., 71 *Enterococcus* spp., and 69 *E. coli* isolates. In *E. coli*, ceftriaxone MIC_50_ and MIC_90_ values were 0.031 and 512 µg/mL, respectively. Five assemblies contained *bla*_CTX-M-1_-labelled intervals that were identical across 876 bp and corresponded to ceftriaxone MICs of 32–1024 µg/mL. In *E. coli* isolate 1316, seven resistance-gene calls co-occurred within a 12,933-bp region of one contig. Assembly quality varied substantially, and the draft assembly of *E. coli* isolate 832 was unsuitable for reliable gene-negative interpretation. **Conclusions**: The dataset showed heterogeneous susceptibility profiles and identifiable resistance-gene contexts. These findings support isolate-linked phenotypic and genomic surveillance while avoiding population-level, transmission, or clinical resistance classifications unsupported by the available data.

## 1. Introduction

Antimicrobial resistance (AMR) in food-producing animals is both an animal-health concern and a component of the wider human–animal–environment interface. Bacterial populations associated with livestock are exposed to antimicrobial treatment, production-related ecological pressures, and opportunities for genetic exchange. Consequently, susceptibility measurements in commensal organisms can contribute information that is not captured by monitoring clinical pathogens alone [1,2]. The scale of antimicrobial use in food production further underlines the importance of preserving interpretable surveillance data, although antibiotic consumption cannot be inferred directly from resistance measurements in an individual collection [3,4].

Commensal *Escherichia coli* is particularly useful for this purpose because susceptibility profiles can be measured with standardized laboratory methods and compared across appropriately harmonized surveillance systems. European monitoring illustrates the value of following indicator organisms over time rather than treating each dataset as an isolated estimate [5]. Gram-positive indicators provide complementary information. Enterococci and staphylococci differ from *E. coli* in their ecological distributions, intrinsic susceptibilities, and clinically important resistance mechanisms; their inclusion therefore broadens surveillance, but also requires organism-specific interpretation [6,7,8]. A high MIC in one bacterial group cannot automatically be interpreted as the same biological event as an equally high value in another. These three bacterial groups were therefore included as complementary commensal indicators within the established Hungarian poultry AMR surveillance framework: *E. coli* provides a Gram-negative indicator, whereas enterococci and staphylococci provide Gram-positive comparators with distinct ecological distributions and intrinsic susceptibility backgrounds. Assembly-level genomic data were available only for the *E. coli* subset; consequently, the genomic analyses were restricted to *E. coli* rather than extended to the Gram-positive isolates.

Hungarian poultry-associated AMR has already been investigated through national susceptibility studies of *E. coli* and *Enterococcus* and through regional analyses within the same research programme [9,10,11]. Genomic work on isolates from Hungarian layers provides an additional foundation for examining resistance determinants [12]. The present study is situated within that programme and does not describe a new, independent national sampling campaign. Its novel contribution lies not in geographic subdivision alone, but in linking the Northern Hungary isolate-level MIC dataset to the available *E. coli* draft assemblies, thereby enabling direct verification of selected resistance-gene intervals and examination of their local sequence context. This provides an assembly-aware genomic layer to an established regional phenotypic surveillance framework while explicitly distinguishing directly supported positive sequence evidence from uncertain negative genomic inference.

The production context also matters. Adult egg-producing chickens should not be conflated with broilers, and bacterial populations collected from tracheal and cloacal samples need not have equivalent susceptibility distributions. Studies in Spanish laying hens and in Austrian broilers illustrate the diversity of production and sampling frameworks used in poultry AMR research [13,14]. Comparisons across such studies are informative only when the organisms, specimen types, antimicrobial panels, and interpretation rules are considered together. Regional descriptions can therefore add value by preserving the relationship between isolate metadata and laboratory measurements rather than relying exclusively on a single multidrug-resistance percentage.

Susceptibility and sequence data answer related but distinct questions. MIC measurements describe growth inhibition under the assay conditions. Detection of a resistance-associated sequence provides evidence that a corresponding determinant is present in the assembled material, but does not by itself establish expression, phenotypic penetrance, or the absence of other mechanisms. Conversely, a missing gene call can reflect incomplete recovery, alignment settings, or database coverage rather than true gene absence. The breadth of curated AMR databases makes them valuable resources, while simultaneously requiring distinctions between acquired determinants, intrinsic loci, and regulatory genes [15].

Two further distinctions are essential when assemblies are fragmented or potentially mixed. First, a gene sequence found in material labelled as an *E. coli* isolate is not necessarily assignable to an *E. coli* chromosome if taxonomic purity has not been established. Second, a sequence-composition classifier that predicts plasmid origin does not replace reconstruction of a complete plasmid or experimental transfer testing [16]. Published investigations of farm animal *E. coli* that explicitly examine resistance genes and mobile genetic elements provide a useful comparison for this hierarchy of evidence [17]. Food-chain studies, including investigations of extended-spectrum β-lactamase-producing *E. coli* in table eggs, further demonstrate why these distinctions matter beyond the farm [18].

The wider Hungarian literature also emphasizes the environmental and information-management dimensions of AMR. A recent review of wild birds describes possible reservoirs and dissemination routes at the wildlife–livestock interface, without making any particular flock an established source or recipient [19]. Work on veterinary public health data infrastructure highlights the value of maintaining traceable links between heterogeneous laboratory and metadata sources [20]. Both perspectives favor an analysis in which specimen identity, measured phenotype, assembly characteristics, and the evidential status of genomic annotations remain visible.

Accordingly, this study had three connected objectives: to describe the MIC distributions of chicken-associated *Staphylococcus*, *Enterococcus*, and *E. coli* collected in Northern Hungary; to characterize the available *E. coli* assembly subset without assuming that it represented the entire regional population; and to examine whether selected resistance-gene calls could be verified on the contigs and placed in a meaningful sequence context. The analysis was restricted to the sampled collection. It was not designed to estimate farm-level risk, infer transmission between holdings, or assign clinical resistance categories where the required interpretive evidence was unavailable.

## 2. Results

### 2.1. Regional Collection and Linkage of the Genomic Subset

The filtered collection contained 166 isolates: 26 *Staphylococcus*, 71 *Enterococcus*, and 69 *E. coli*. All records identified the host as domestic chicken, the production purpose as egg production, and the age category as adult. There were 84 tracheal and 82 cloacal isolates in total. Specimen composition differed between bacterial groups: all 26 staphylococci were tracheal, whereas enterococci comprised 35 tracheal and 36 cloacal isolates, and *E. coli* comprised 23 tracheal and 46 cloacal isolates. These differences are relevant to descriptive cross-group comparisons and were not removed by statistical adjustment (Table 1; Supplementary File S1). Species assignments further characterized the Gram-positive subsets. All 26 staphylococcal isolates were identified as *Staphylococcus aureus*. Among the 71 enterococci, the recorded assignments comprised *Enterococcus durans/hirae* (13/71; 18.3%), *E. faecium* (4/71; 5.6%), *E. pseudoavium* (2/71; 2.8%), *E. columbae* (1/71; 1.4%), and *E. saccharolyticus* (1/71; 1.4%); the remaining 50 isolates (70.4%) were retained as *Enterococcus* spp. because an unambiguous species-level assignment was not available; isolate-level taxonomic assignments are provided in Supplementary File S1. Thus, the staphylococcal subset was taxonomically uniform at species level, whereas the enterococcal subset remained heterogeneous and incompletely resolved at species level.

All 24 assembly filenames could be linked unambiguously to a chicken-origin, Northern Hungary *E. coli* record using the combination of bacterial group and isolate number. These assemblies covered 24/69 isolates (34.8%). The sequenced subset contained 22 cloacal and two tracheal isolates, compared with 24 cloacal and 21 tracheal isolates among the 45 records without an assembly. Fourteen assembly-linked isolates originated from 2022 and ten from 2023; the corresponding counts among the non-sequenced records were 35 and 10. Genome availability was therefore neither specimen-balanced nor proportional to the sampling-year distribution (Supplementary File S1; Figure S4).

The full *E. coli* collection and the assembly-linked subset were retained as separate analytical denominators throughout. No additional genomes from other poultry species were added. The 24 assembly identifiers remained linked to their original MIC measurements, annotation coordinates, and sequence files, including the highly fragmented assembly of isolate 832. Locality spelling was standardized where supported by an explicit source entry, but locality was not used as a grouping factor or predictor.

### 2.2. Recorded MIC Distributions Differed by Organism and Antimicrobial

**Figure 1:**
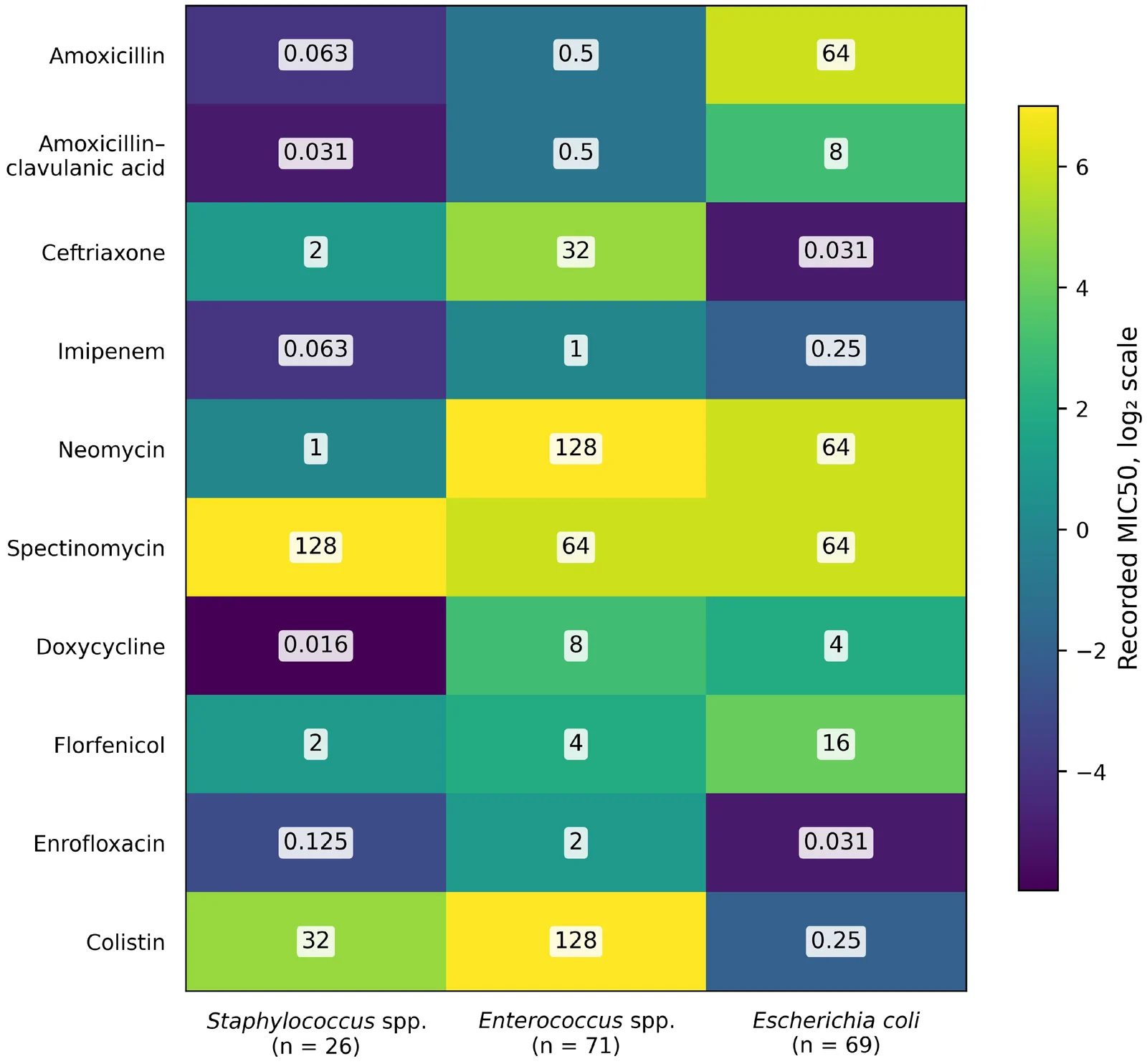
Recorded MIC_50_ values for the ten antimicrobials included in the cross-group visualization. Complete 15-agent minimum inhibitory concentration (MIC) distributions are provided in Supplementary File S1. Numbers are in µg/mL; color intensity represents log2-transformed MIC_50_. These are measured distributions, not clinical susceptibility categories. Colistin values in Gram-positive organisms and ceftriaxone values in enterococci are retained as recorded observations, not evidence of acquired resistance. Genus and specimen composition differ, precluding direct comparisons of resistance risk. MIC–minimum inhibitory concentration.

The complete data matrix comprised 2490 recorded isolate–antimicrobial combinations across the 15 tested agents. No values were imputed. The MIC_50_ and MIC_90_ summaries describe the ordered measurements as recorded in the source workbook, rather than interpolated concentration values or inferred clinical categories (Table 2). Selected profiles are shown in Figure 1, and the full distributions, including the frequency of the upper-end entry 1024, are provided in Supplementary File S1.

In *E. coli*, amoxicillin MIC_50_/MIC_90_ values were 64/512 µg/mL, whereas the corresponding amoxicillin–clavulanic acid values were 8/128 µg/mL. Ceftriaxone displayed a particularly pronounced difference between its median and upper distribution: the recorded MIC_50_ was 0.031 µg/mL, and the MIC_90_ was 512 µg/mL. Five ceftriaxone records were entered as 1024. Thus, the low median did not imply a uniformly low-MIC population. Imipenem MIC_50_/MIC_90_ values were 0.25/1 µg/mL, providing a contrasting β-lactam profile within the same set of isolates.

The *E. coli* distributions for doxycycline and florfenicol also had broad upper tails, with MIC_50_/MIC_90_ values of 4/256 and 16/512 µg/mL, respectively. Neomycin values were centered at 64 µg/mL, with an MIC_90_ of 128 µg/mL, while spectinomycin values were 64/512 µg/mL. Enrofloxacin had a recorded MIC_50_ of 0.031 µg/mL and an MIC_90_ of 2 µg/mL. Colistin had a low median of 0.25 µg/mL, but its MIC_90_ coincided with the upper-end entry 1024; seven isolates had that entry. Because the coding of off-scale results could not be reconstructed independently, these values are reported as recorded rather than interpreted as exact validated endpoints.

Enterococci showed amoxicillin and amoxicillin–clavulanic acid MIC_50_/MIC_90_ values of 0.5/2 µg/mL for both agents. Doxycycline values were 8/32 µg/mL and enrofloxacin values were 2/32 µg/mL. The neomycin distribution was shifted towards high recorded values, with MIC_50_/MIC_90_ values of 128/1024 µg/mL. Ceftriaxone and colistin measurements are retained in the complete data record, but high values for these agents in enterococci are not interpreted as newly acquired resistance phenotypes. The enterococcal dataset comprised several recorded species or species-pair assignments together with isolates retained only at the genus level, which further limits application of a single species-specific categorical interpretation scheme.

Staphylococci had lower median values for several agents, including amoxicillin (0.063 µg/mL), doxycycline (0.016 µg/mL), and enrofloxacin (0.125 µg/mL). The corresponding MIC_90_ values were 0.125, 4, and 16 µg/mL. The separation between low median and higher upper-tail values was therefore not confined to *E. coli*. However, the staphylococcal sample was smaller and exclusively tracheal; therefore, these measurements do not establish that its resistance burden was lower than that of either of the other bacterial groups.

### 2.3. Descriptive Covariation and Multivariate Structure of the MIC Profiles

**Figure 2:**
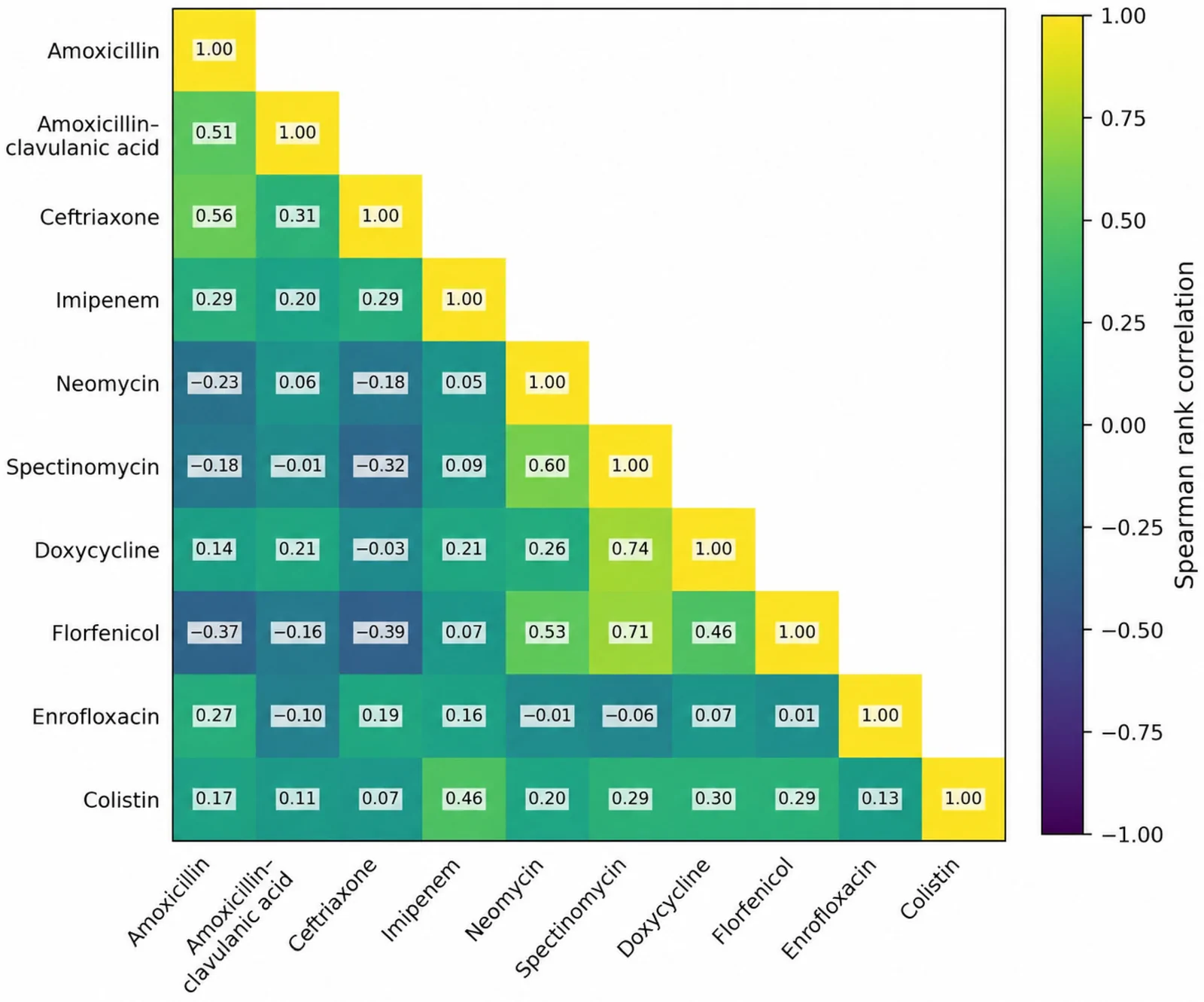
Descriptive pairwise Spearman correlations of recorded MICs in *Escherichia coli*. Coefficients were recalculated from the isolate-level MIC data, without dichotomizing them at unvalidated thresholds. Upper-triangle cells are suppressed. The matrix does not establish genetic linkage or farm-adjusted statistical significance. MIC–minimum inhibitory concentration.

Pairwise correlations were calculated directly from isolate-level MIC measurements. In *E. coli*, the strongest positive relationships among the tested measurements included spectinomycin–doxycycline (Spearman ρ = 0.735), spectinomycin–florfenicol (ρ = 0.708), and neomycin–spectinomycin (ρ = 0.604). Amoxicillin and ceftriaxone also covaried positively (ρ = 0.557). These coefficients describe the collection-level co-distribution of MIC ranks; they are not evidence that the underlying determinants occur on the same genetic element (Figure 2; Supplementary File S1).

In enterococci, a strong positive correlation was observed between amoxicillin and amoxicillin–clavulanic acid (ρ = 0.876), as were the relationships of amoxicillin and amoxicillin–clavulanic acid with imipenem (ρ = 0.756 and 0.751, respectively). Doxycycline–enrofloxacin covariation was also positive (ρ = 0.613). Among staphylococci, tylosin–enrofloxacin showed ρ = 0.622 and trimethoprim–sulfamethoxazole–vancomycin showed ρ = 0.562. The corresponding matrices are provided as Figures S1 and S2. They should be read as descriptive patterns in mixed, specimen-dependent collections rather than as tests of common molecular mechanisms.

**Figure 3:**
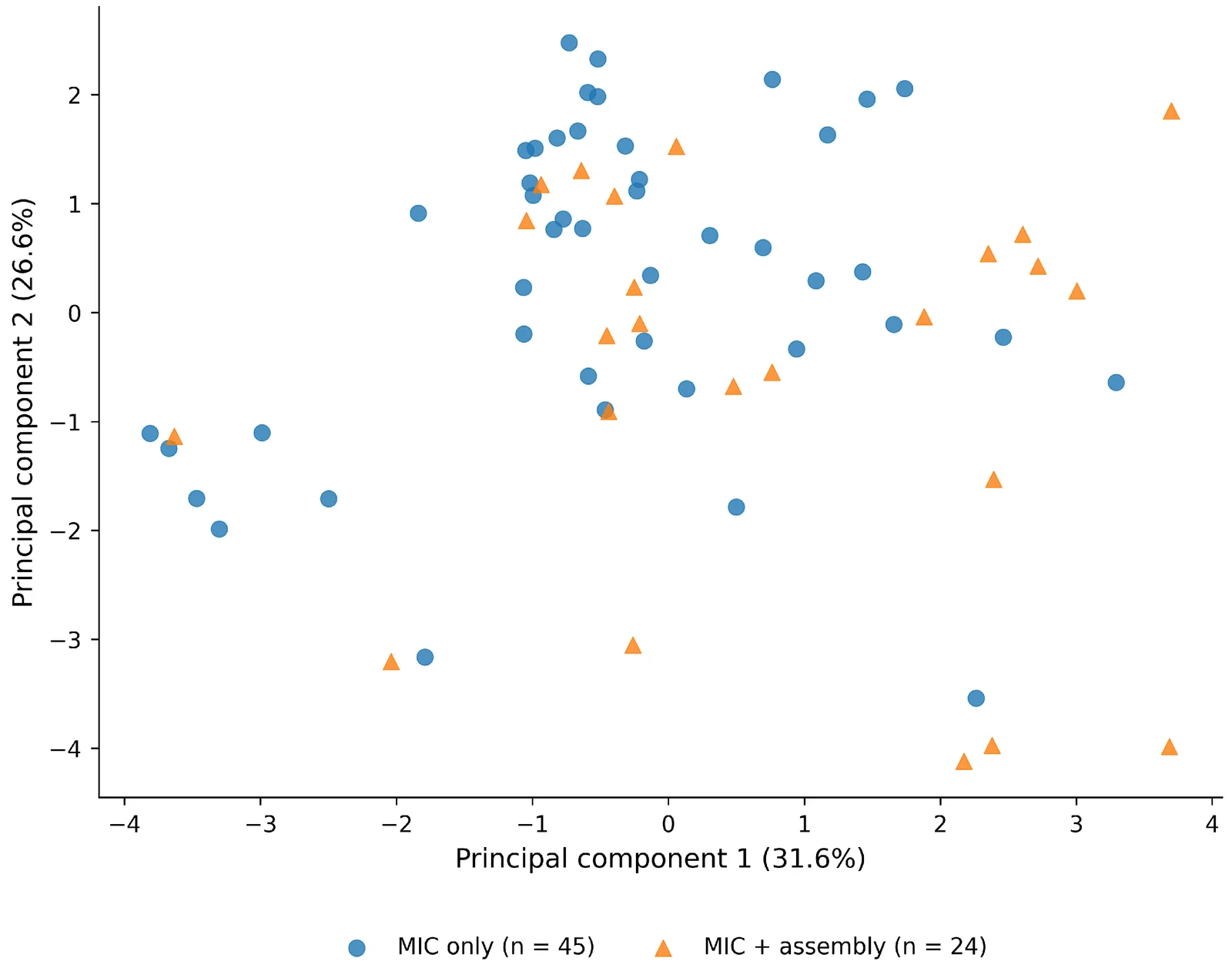
Principal component projection of the 69 *Escherichia coli* minimum inhibitory concentration (MIC) profiles. Ten drugs from the original *E. coli* analytical panel were log2-transformed, centered, and scaled to unit population variance before singular-value decomposition. All 69 observations are plotted; identical profiles can overlap. Marker shape indicates availability of an assembly, not a phenotypic or genomic cluster. The axes explain 31.6% and 26.6% of the variance, respectively.

Principal component analysis of the standardized, log2-transformed ten-agent *E. coli* panel explained 31.6% of the variance on the first axis and 26.6% on the second (Figure 3). Together, the first two components represented 58.2% of the observed multivariate variation. The projection visualized heterogeneity among all 69 isolates without imposing a prespecified number of clusters. The 24 assembly-linked records were marked separately, but the plot was not used to claim unbiased selection or define genomic lineages. Coordinates and loadings are in Supplementary File S1, and the individual MIC profiles are shown in Figure S3.

An exploratory sensitivity analysis using the numeric thresholds recorded in the regional dataset yielded threshold-positive class counts. Under the explicitly documented study-panel class mapping, at least three threshold-positive classes occurred in 21/26 staphylococci, 57/71 enterococci, and 56/69 *E. coli*. These are operational threshold-exceedance counts only. They are confined to the supplementary sensitivity analysis because the source entries do not establish a complete, applicable clinical-breakpoint or epidemiological-cutoff scheme (Supplementary File S1). No XDR or PDR frequencies are inferred from this reconstruction.

### 2.4. Assembly Continuity and Taxonomic Warning Signals

Length-based preparation removed 5987 contigs shorter than 200 bp, representing 596,898 bp across the 24 files. No retained contig was shortened to force an expected genome size, and no artificial sequence was added. After filtering, assembly lengths ranged from 2,850,602 to 11,152,220 bp, with a median N_50_ of 3389 bp and a median of 3818 contigs. The complete before/after metrics, checksums, and contig-identifier mappings are in Supplementary File S1.

**Figure 4:**
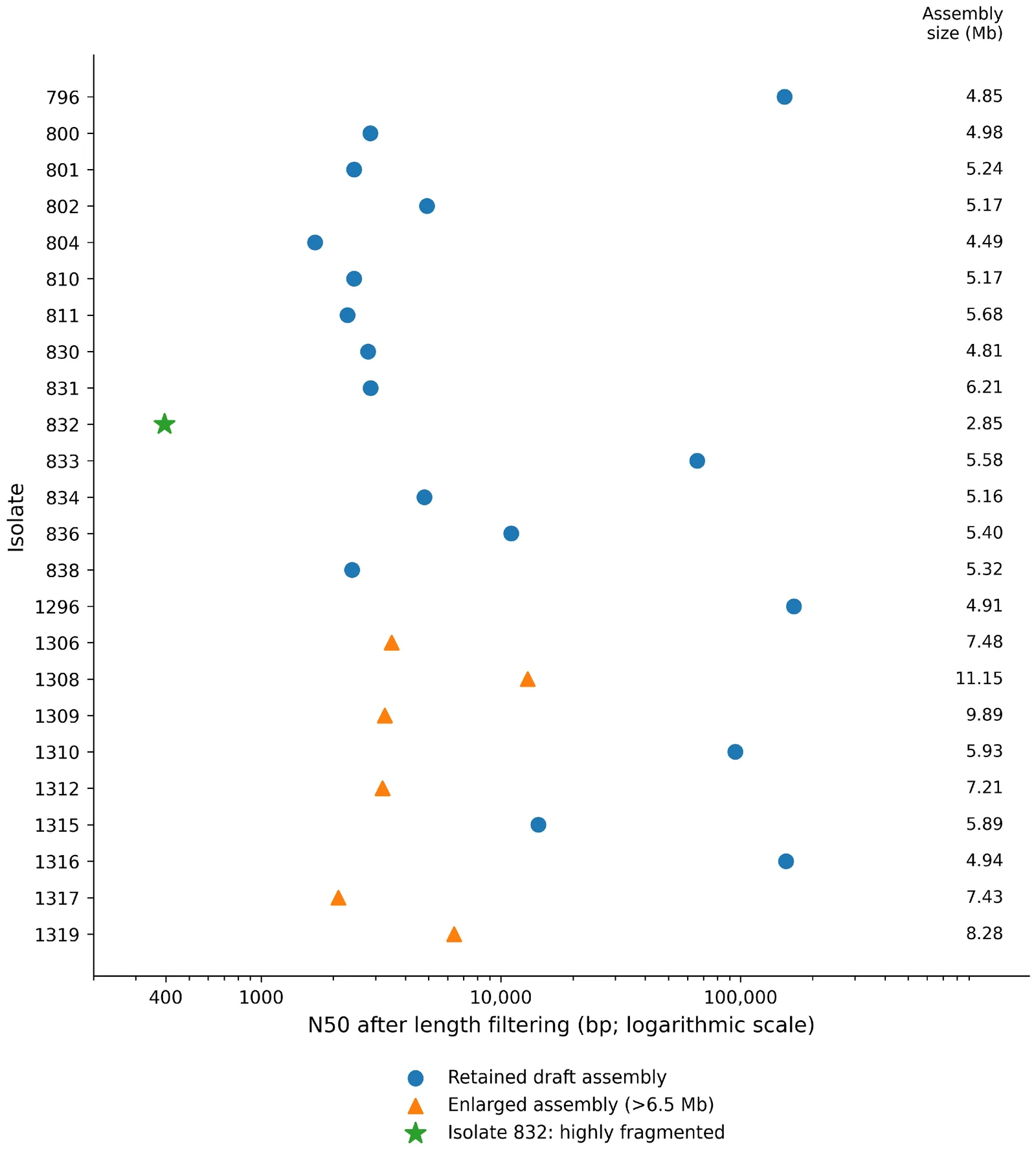
Assembly continuity after the removal of contigs shorter than 200 bp. Each point is one of the 24 retained assemblies, and the right-hand annotation gives the total retained length. The >6.5-Mb flag is a descriptive size warning, not an NCBI acceptance threshold or a taxonomic diagnosis. Isolate 832 remains in the package; its 395-bp N_50_ does not support reliable gene-negative or whole-genome comparative inference. No size-targeted truncation was applied.

Continuity differed markedly among samples. Isolates 796, 1296, and 1316 had filtered N50 values of 152,424, 166,787, and 154,530 bp, respectively. In contrast, the retained assembly for isolate 832 comprised 7470 contigs totaling 2,850,602 bp, with an N_50_ of only 395 bp. Isolate 832 remained in the phenotypic collection, the assembly inventory, and the evidence tables, but its missing gene calls were not treated as reliable evidence of absence (Figure 4).

### 2.5. Resistance-Gene Evidence in the Assembly-Linked Records

The regional subset of the CARD format alignment tables contained 767 reported hits. Every hit could be mapped to a named original contig, and all coordinate intervals remained present after the length filter. Thirty-four identity fields were not interpretable as numerical percentages because of spreadsheet conversion; these were marked unavailable rather than reconstructed from date-like values. The accompanying coverage, reference-accession, and coordinate fields are preserved in Supplementary File S1.

**Figure 5:**
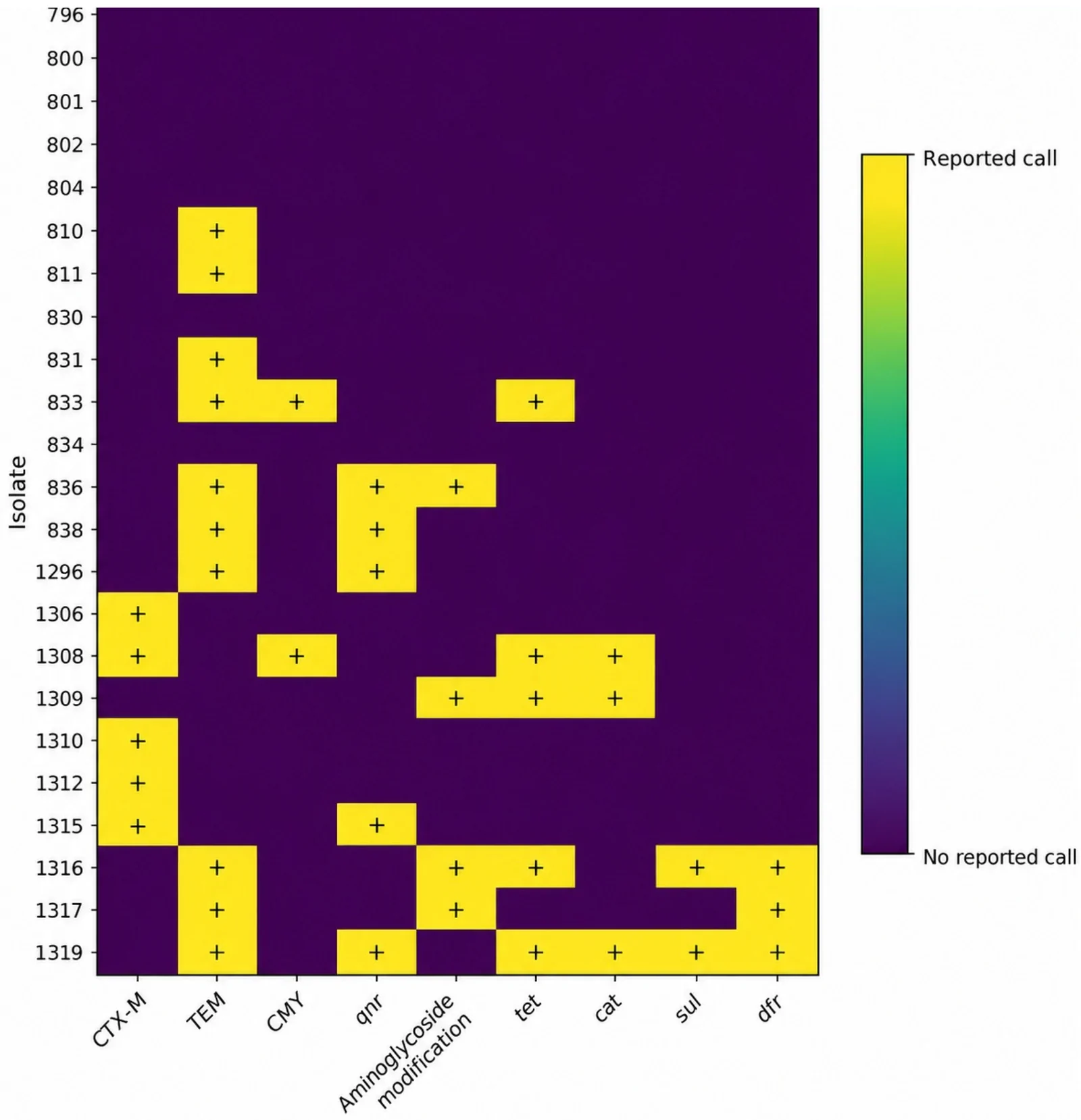
Distribution of selected resistance-gene families among 23 *Escherichia coli* assemblies. Isolate 832 was omitted from this visualization because its highly fragmented assembly did not support reliable interpretation of gene non-detection; it remains in the genomic inventory and phenotypic dataset. Filled cells indicate at least one corresponding CARD-based alignment, whereas unfilled cells indicate no reported alignment, not confirmed gene absence. Gene-level annotations, alignment coverage, sequence identity, and contig coordinates are provided in Supplementary File S1.

The analysis distinguished selected resistance-gene families from the many efflux, regulatory, and intrinsic-associated entries in the source output. TEM-family calls were reported in ten assemblies: eight carried the label *bla*_TEM-1_, one *bla*_TEM-12_, and one *bla*_TEM-35_. Five assemblies carried a *bla*_CTX-M-1_ call, and two carried a *bla*_CMY-59_ call. Other selected calls included *qnrS1* in four assemblies, *tet(A)* in two, and *tet(J)* in three. The complete isolate-level matrix is provided in Supplementary File S1, while Figure 5 summarizes selected families. These frequencies refer to reported calls within the available assembly subset rather than prevalence among all 69 *E. coli* or among chickens in the region.

Absence of a selected call was not equated with a fully susceptible genotype. In particular, isolate 832 contained only a small number of reported regulatory or efflux-associated hits, and its highly fragmented sequence record was classified as unsuitable for negative-evidence interpretation. Likewise, potentially mixed assemblies could contain biologically real AMR sequences whose organism of origin was unresolved. Genomic observations are therefore described as assembly-linked evidence unless a stronger assignment is supported by the data.

### 2.6. Shared CTX-M-Labelled Sequence Intervals and Ceftriaxone MICs

The five CTX-M-1-labelled calls occurred in isolates 1306, 1308, 1310, 1312, and 1315. Each alignment reported 100% reference coverage and 100% nucleotide identity across an 876-bp interval. Direct extraction from the original contigs, with strand correction, showed that all five intervals were identical. The sequence checksums and original-to-renamed contig mappings are available in Supplementary File S1. This verifies a shared sequence at the reported locus; it does not establish whole-genome clonality or identify a shared complete plasmid (Table 3).

**Figure 6:**
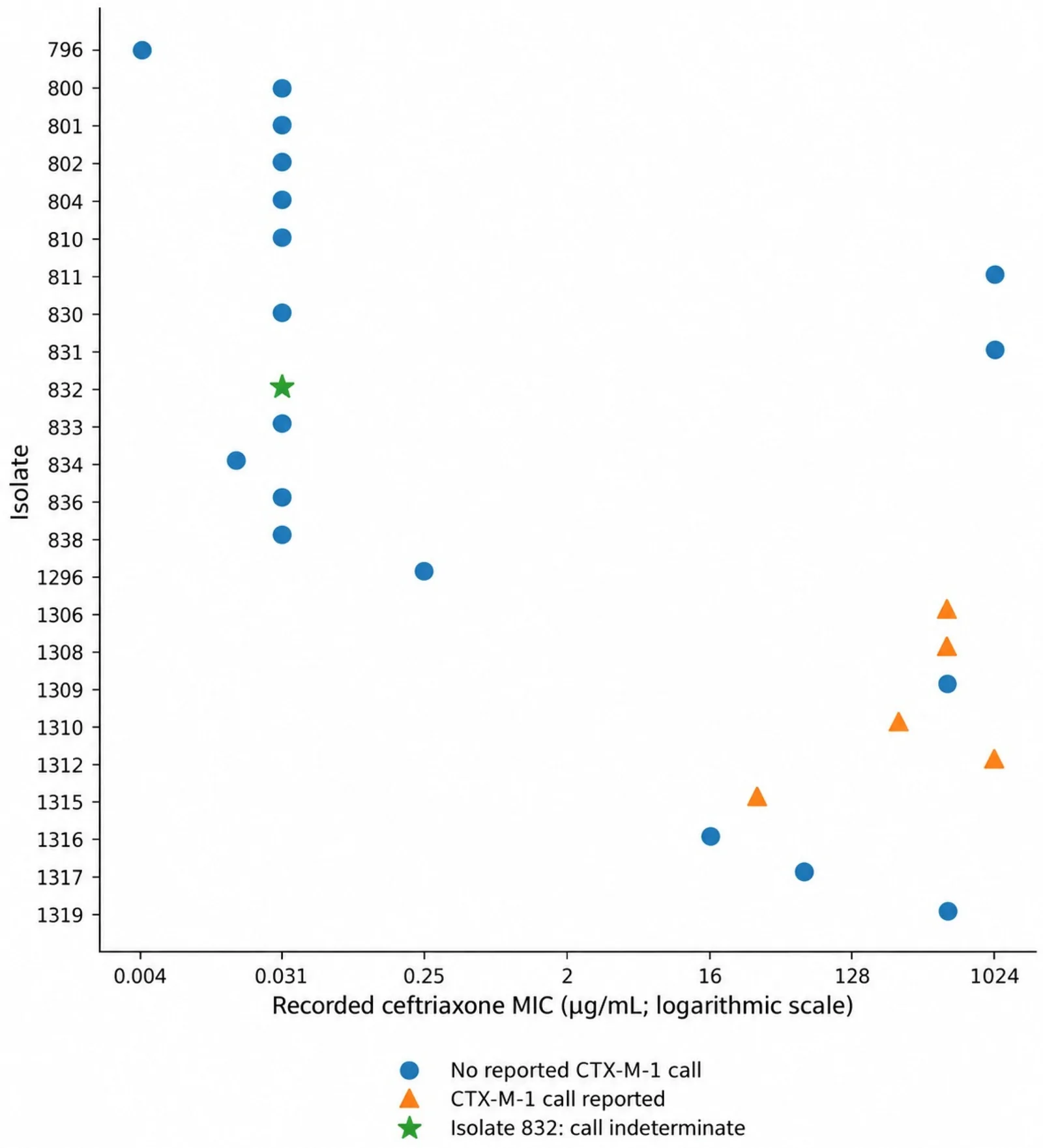
Isolate-level comparison of *bla*_CTX-M-1_ calls and recorded ceftriaxone minimum inhibitory concentrations (MICs). The five call-positive assemblies correspond to MIC entries of 32–1024 µg/mL. Other high-MIC isolates do not have a *bla*_CTX-M-1_ call; no susceptible or gene-absent status is inferred from that fact. The endpoint entry 1024 is preserved as supplied because its off-scale coding cannot be independently reconstructed. No sensitivity, specificity, or extended-spectrum β-lactamase (ESBL) confirmation rate is estimated.

The corresponding recorded ceftriaxone MICs were 512, 512, 256, 1024, and 32 µg/mL, respectively (Figure 6; Supplementary File S1). All five therefore fell within the upper portion of the *E. coli* ceftriaxone distribution. However, other high-MIC records lacked a *bla*_CTX-M-1_ call. The data do not support interpretation of that lack as a false-negative genomic test because the completeness of negative detection and the applicability of a binary clinical reference standard were not established. No diagnostic sensitivity, specificity, or predictive value was calculated.

All five CTX-M-containing contigs were assigned plasmid-associated labels in the PlasFlow results, but the predicted taxonomic labels were inconsistent: two were labelled Proteobacteria, two Firmicutes, and one unclassified. These assignments were retained in the evidence table without translating them into a bacterial host identity. No CTX-M-associated mobile-element annotation was reported in the accompanying local context fields. Consequently, the findings support recovery of the CTX-M-labelled sequence from the assembled material, but not a particular plasmid backbone or transfer route.

### 2.7. A Multi-Gene Resistance Context in Isolate 1316

**Figure 7:**
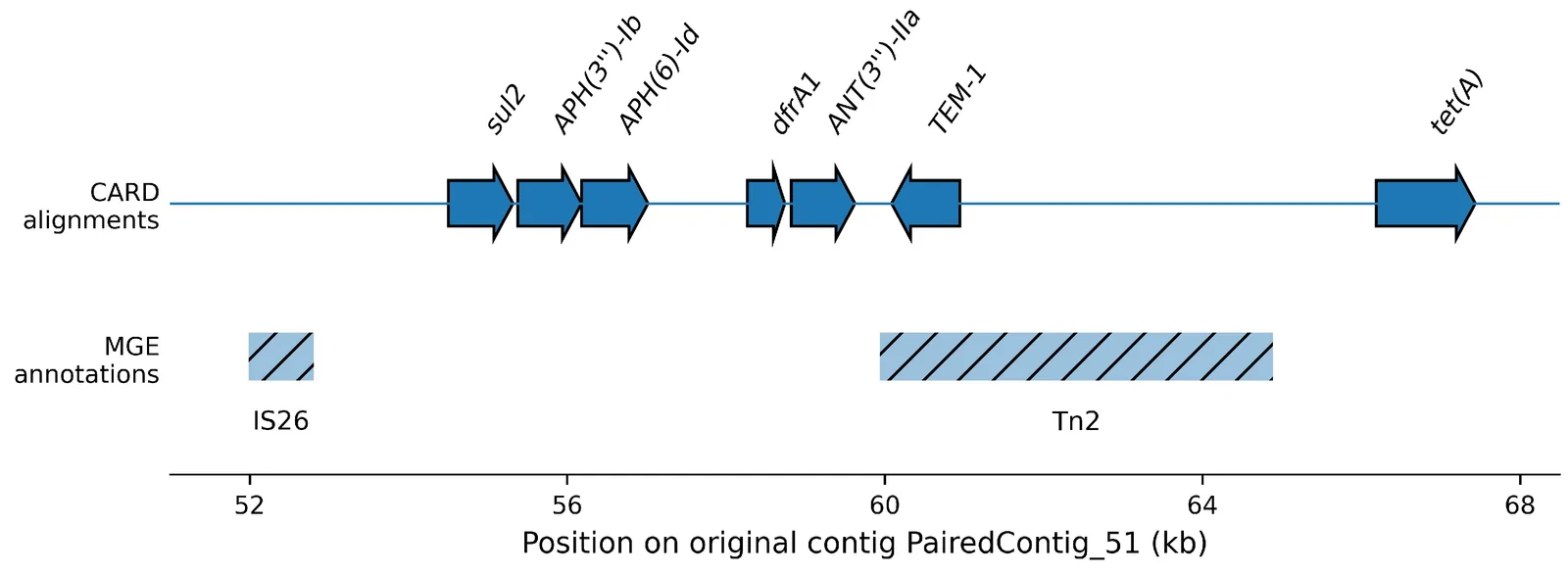
Contig-level co-occurrence of seven resistance-gene alignment calls in isolate 1316. Coordinates and orientation were taken from the original CARD format output and verified against the retained sequence of PairedContig_51. Hatched rectangles show the IS26 and Tn2 annotations. The *bla*_TEM_-family call lies inside the reported Tn2 interval; *tet(A)* lies downstream. The context is an assembled contig with a classifier-derived plasmid label, not a closed plasmid, an independently validated transposon, or a demonstrated transfer event. Abbreviations: CARD, Comprehensive Antibiotic Resistance Database; MGE, Mobile Genetic Elements.

Isolate 1316 provided a more contiguous example for examining local gene organization. Seven reported resistance-gene intervals occurred on the same original contig, *PairedContig_51*, renamed EC1316_ctg00050 during FASTA preparation. From the first to the last reported gene coordinate, they occupied a 12,933-bp span. The calls comprised *sul2*, APH(3″)-Ib, APH(6)-Id, *dfrA1*, ANT(3″)-IIa, a TEM-family β-lactamase, and *tet(A)*. Their positions and strand orientations are shown in Table 4 and Figure 7.

The mobile-element output annotated an IS26 interval upstream and a Tn2 interval spanning positions 59,937–64,886. The TEM-labelled interval at positions 60,084–60,944 lay within that reported Tn2 interval, whereas *tet(A)* at positions 66,190–67,436 lay downstream. This spatial distinction was preserved: the entire seven-gene region was not described as a single experimentally resolved transposon. The contig had a plasmid-associated sequence-composition label, but no closed plasmid sequence or transfer experiment was available.

These observations establish co-occurrence of the reported gene intervals on an assembled sequence and provide a concrete local explanation for how several resistance determinants could be carried together. They do not demonstrate that every associated phenotype was expressed, that the assembly junctions were independently validated by read mapping, or that the element moved between isolates. The relevant annotations, extracted-sequence identifiers, checksums, and coordinate checks are provided in Supplementary File S1 to make these boundaries of interpretation explicit.

## 3. Discussion

This study integrates a multi-organism susceptibility dataset with assembly-linked evidence from a subset of *E. coli*. Its main biological findings are the coexistence of low central MIC values and pronounced upper tails for selected agents, recovery of an identical CTX-M-1-labelled interval in five assemblies, and a multi-gene resistance context in isolate 1316. Together, they provide a more resolved description of the sampled collection than a single categorical MDR percentage. The inference remains at the level of the available isolates and sequences, not all commercial chicken production in Northern Hungary.

The ceftriaxone distribution illustrates why reporting both central tendency and the upper distribution is important. Its very low *E. coli* MIC_50_ coexisted with a recorded MIC_90_ of 512 µg/mL and with five assemblies carrying the same CTX-M-labelled interval. A median alone would have obscured this subpopulation. Conversely, the high upper-tail entries do not justify assigning a clinical resistance category without a documented interpretive framework. Maintaining the complete dilution-level data allows the collection to be reinterpreted when an applicable, versioned scheme is available, rather than locking the analysis to uncertain retrospective labels.

The broad distributions for doxycycline, florfenicol, and selected aminoglycoside-related agents similarly indicate heterogeneity within the collection. International poultry studies provide relevant context for these classes, but their percentages should not be treated as directly interchangeable with the present measurements because production type, specimen origin, and interpretation vary [2,13,14]. The current records specifically represent adult egg-producing chickens. They should not be discussed as if they were a random sample of broiler caecal flora or slaughterhouse isolates.

The Gram-positive findings further demonstrate the need for species-level biological context. The enterococcal collection comprised multiple recorded taxa together with a substantial proportion of isolates that could be retained only at the genus level. Because intrinsic and acquired susceptibility patterns can differ among *Enterococcus* species, the pooled genus-level MIC distributions should not be interpreted as species-specific susceptibility estimates or as direct indicators of a species-level clinical risk. Earlier studies of broiler-breeder *E. faecalis* and enterococci from different farming systems likewise illustrate why species composition and sampling design matter [21,22]. The staphylococcal subset presents a different limitation: although all 26 isolates were identified as *S. aureus*, species identification alone does not establish methicillin resistance, pathogenicity, or an equivalent clinical risk [8,23]. Furthermore, this subset was smaller and exclusively tracheal, precluding an unqualified cross-group ranking of resistance burden.

The five identical 876-bp *bla*_CTX-M-1_-labelled intervals constitute positive sequence evidence beyond a gene-name summary. They were extracted directly from the contigs and could be matched across isolates after accounting for strand orientation. Their correspondence with high recorded ceftriaxone MICs is biologically consistent with the resistance-associated annotation, while the interpretation does not require all assemblies to be sufficiently complete for whole-genome comparison. Importantly, identical gene sequence is a narrower conclusion than identical lineage or a shared transmission event.

The occurrence of additional high ceftriaxone MICs without a corresponding CTX-M call is also informative, but not diagnostically decisive. Possible explanations include other β-lactamases, chromosomal regulatory variations, incomplete sequence recovery, annotation limitations, or laboratory-data uncertainty. The present data cannot distinguish these alternatives comprehensively. TEM-family calls with less than perfect sequence identity were therefore not promoted to definitive allele assignments, and the presence of a generic chromosomal AmpC-associated label was not treated as proof of acquired ESBL activity. Curated resistance databases facilitate detection and interpretation, but homology calls still have to be connected to the specific sequence, organism, and phenotype [15].

The local context in isolate 1316 adds a different type of information. Several reported resistance determinants occupied a single contig, with a TEM-family interval lying inside a Tn2 annotation and *tet(A)* downstream. This provides an assembly-level example of co-carriage that is stronger than phenotypic correlation alone. At the same time, the source annotations do not establish the integrity, mobility, or host range of that element. Studies that combine gene detection with investigation of mobile elements are therefore methodologically relevant, while experiments demonstrating transfer remain distinct from our sequence-based observations [17].

The food-chain significance of such findings is plausible but must remain contextual. Detection of ESBL-producing *E. coli* in table eggs from Istanbul connects resistance surveillance to a food commodity, whereas the present study sampled living chickens and did not examine eggs or consumers [18]. Likewise, the previous resistome study in Hungarian layers establishes a relevant national research context but does not make the current 24 assemblies a representative layer resistome survey [12]. The defensible contribution here is an isolate-linked description of the available Northern Hungary material, with stronger claims limited to directly recoverable sequence observations.

A central distinction in this dataset is between positive sequence evidence and negative genomic inference. Even a fragmented assembly can contain a recoverable resistance-gene interval. However, failure to detect a gene in such an assembly cannot be interpreted with the same confidence as its successful recovery. Isolate 832 illustrates this asymmetry: retaining the isolate preserves its measured phenotype and sample identity, but its 395-bp N50 and short total assembly length leave a large uncertainty around gene absence and genome-wide relationships. Retention is not equivalent to declaring the genome complete.

Long assemblies and taxonomic warning signals create a different uncertainty. In several cases, inflated sequence length coincided with non-target species-rank entries in the taxonomic summaries. Without contig-level assignments or underlying reads, it would have been unjustified to remove sequences merely because they carried a gene commonly associated with another organism. Such a rule could delete a legitimate mobile determinant. It would have been equally unjustified to label all retained sequences as confirmed *E. coli*. Accordingly, sequence ownership remains unresolved where the evidence is insufficient.

The classifier-derived plasmid labels also require restraint. PlasFlow uses sequence signatures to predict origin and is useful for exploring fragmented data, but it does not close plasmids or establish taxonomic host identity [16]. The inconsistent phylum labels among the CTX-M-containing contigs are a concrete reason not to convert those predictions into claims about cross-phylum transmission. More generally, aggregate Kraken 2/Pavian outputs are informative screening summaries, not substitutes for coordinate-level evidence about which segment should be removed [24,25].

Assembly statistics should therefore be interpreted as indicators of information availability rather than a certificate of correctness. The contrast between high-continuity samples such as 1316 and highly fragmented samples such as 832 determines which analyses are supportable. Continuity metrics are useful components of genome-quality assessment, but neither total length nor N50 alone establishes completeness, purity, or correct assembly [26]. For the same reason, removal of sub-200-bp sequences is a file-preparation step and must not be presented as complete decontamination or guaranteed acceptance by a sequence repository [27].

The present study extends an established Hungarian poultry antimicrobial-resistance surveillance programme by providing a regionally resolved Northern Hungary dataset within the same standardized framework used in previous national and regional investigations [9,10,11]. Its principal added value lies in the integration of isolate-level MIC measurements with assembly-linked genomic evidence, including coordinate-verified resistance-gene calls and sequence-context analysis. This combined approach increases the molecular resolution of the regional surveillance data while preserving a clear distinction between directly supported genomic observations and uncertain negative evidence.

Because clinical breakpoints, epidemiological cutoff values, and study-specific numeric thresholds are not interchangeable, the primary analysis focused on measured MIC distributions. The current EUCAST definitions explicitly distinguish susceptible, susceptible with increased exposure, and resistant categories, while the MDR expert proposal depends on valid antimicrobial category assignments [28,29]. A retrospective binary matrix assembled from uncertain or mixed rules cannot be made reliable by applying additional clustering algorithms. The primary analysis therefore preserves the measured MIC profiles, with a separately labelled threshold sensitivity analysis for traceability.

The same principle applies to apparent phenotypic clusters. Correlation and principal component projection can describe structured variations in the observed data, but they do not identify bacterial clones, shared resistance plasmids, or causal exposure histories. By avoiding a forced three-cluster partition and an MDR classifier trained on the variables defining its own outcome, the analysis gives priority to directly interpretable information. In this collection, the sequence context in isolate 1316 is more informative about physical co-carriage than a dense network of raw co-resistance counts would be.

The findings support preservation of linked regional records for surveillance and stewardship, not an empirical treatment recommendation for individual flocks. Antimicrobial-use records would be needed to examine exposure–response relationships. Studies that directly collect poultry antimicrobial-use data illustrate that requirement [30]. Recent Hungarian work quantifying changes in antibiotic use on pig farms provides a relevant example of consumption data collection, but those pig-sector observations cannot be used as a surrogate for exposure in the chicken flocks sampled here [31].

Prevention is likewise broader than the susceptibility of the recovered isolates. Environmental interfaces, including possible contacts with wild birds, warrant attention in biosecurity discussions, but no wildlife source attribution was attempted in this study [19]. A recent Hungarian review of stabilized hypochlorous acid considers disinfection within modern hygiene; it is relevant to the prevention framework rather than evidence that a particular disinfectant reduced AMR in the present collection [32]. The distinction between a prevention option discussed in the literature and an intervention evaluated experimentally should remain explicit.

Antimicrobial peptide research offers another preventive and therapeutic-development context. Hungarian reviews discuss both their broader anti-infective potential and applications aimed at maintaining poultry gut health [33,34]. These approaches were not tested here, and their mention does not imply efficacy against the isolates in this dataset. Instead, the regional susceptibility and sequence records can help define the organisms and resistance contexts against which future interventions should be assessed with appropriate experimental controls.

Finally, the analysis demonstrates a practical data management requirement. Isolate names, specimen descriptors, MIC values, contig identifiers, and annotation coordinates must remain linked, even when reports originate from different processing batches. Work on veterinary public health data infrastructure provides an appropriate framework for that integration [20]. In the present package, explicit keys, unmodified measured values, old-to-new contig mappings, and sequence checksums preserve an auditable path from source material to figures. This does not recover missing laboratory information, but it prevents additional uncertainty from being introduced during data integration.

The collection was not a probability sample of all chicken flocks in Northern Hungary, and the available data did not support adjustment for farm clustering. The results are therefore descriptions of sampled isolates, not population-level regional prevalence estimates. The genomic subset was strongly enriched for cloacal records: 22/24 assembly-linked *E. coli* isolates were cloacal, compared with 24/45 among the non-sequenced *E. coli* isolates. Because only two tracheal isolates were represented in the genomic subset and the historical mechanism governing sequencing availability was not documented, no meaningful comparison of genomic features by specimen type was possible. Consequently, the observed recovery and apparent frequencies of specific resistance-gene fragments, plasmid-associated sequence labels, and other assembly-linked genomic features may partly reflect the source composition of the sequenced subset and should not be interpreted as specimen-independent patterns. These limitations preclude causal comparisons between specimen types and population-level extrapolation of the genomic findings.

Laboratory interpretation was constrained by incomplete provenance for the categorical thresholds and upper-end MIC coding. Genomic inference in the present downstream reanalysis was constrained by the unavailability of the original raw-read files, variable assembly continuity, taxonomic warning signals, and damaged numerical fields in some annotation tables. No complete plasmid reconstruction, phylogenetic transmission analysis, pathotype assignment, or comprehensive mutation-based genotype prediction was performed. These omissions are reflected in the claims made, rather than filled by extrapolation from gene names.

Nevertheless, the study retains useful positive evidence: complete isolate-level susceptibility entries, explicit assembly–phenotype linkage, recoverable CTX-M-labelled sequences, and a traceable multi-gene contig context. The central conclusion is therefore not that every resistance mechanism has been resolved, but that the available regional material supports a bounded, integrated analysis whose evidential strengths and weaknesses can be inspected at isolate level.

## 4. Materials and Methods

### 4.1. Study Design and Sample Collection

This observational laboratory study investigated commensal bacterial isolates obtained from large-scale chicken flocks in Northern Hungary between 2022 and 2023. Sampling was performed on three commercial poultry farms in Northern Hungary during routine veterinary diagnostic visits. Available locality metadata identified Szendrő and Kazincbarcika, although locality information was not available for every source record. Farm identities were anonymized, and exact farm addresses and GPS coordinates were not part of the analytical dataset; consequently, exact geospatial coordinates cannot be reported retrospectively. Metadata retained for epidemiological characterization included the sample type, production purpose, age category, and flock-size category. Breed or commercial genetic-line information was not consistently recorded in the source diagnostic metadata and was therefore not available for analysis.

Tracheal and cloacal swab samples were collected by the attending veterinarians during routine diagnostic visits using sterile Amies-type transport swabs without charcoal. Individual birds were not selected according to a research-defined random or probabilistic sampling scheme. Bird selection was undertaken by the attending veterinarians in the context of routine diagnostic visits according to the diagnostic needs of the flock; the present study subsequently analyzed the bacterial isolates recovered from those diagnostic samples. Bacterial isolates recovered from these samples constituted the analytical units of the present study; consequently, the isolate counts should not be interpreted as the numbers of sampled birds or individual swabs. The analytical dataset contained at most one isolate per target bacterial group for a given source sample; consequently, a single source sample could contribute isolates belonging to more than one bacterial group. Sampling was restricted to routine diagnostic procedures and did not involve experimental infection, treatment allocation, invasive intervention, or any research-driven manipulation of the animals. Following collection, samples were transported to the Microbiology Laboratory of the Department of Pharmacology and Toxicology, University of Veterinary Medicine Budapest, Hungary, for bacterial isolation and antimicrobial susceptibility testing. All microbiological procedures were performed in the institutional microbiology laboratory under the established biosafety and biosecurity procedures applicable to routine bacterial culture and antimicrobial susceptibility testing. No separate study-specific biosafety or biosecurity approval was required for these laboratory procedures.

This study was evaluated by the Animal Welfare Committee of the University of Veterinary Medicine Budapest, which confirmed that the work did not constitute an animal experiment under Act XXVIII of 1998 on Animal Protection and Government Decree No. 40 of 2013 on animal experiments. Because sampling was limited to routine diagnostic tracheal and cloacal swab collection performed by veterinarians attending the farms, notification to or authorization by the Animal Welfare Authority was not required. A formal certificate of exemption for the study was issued by the Animal Welfare Committee on 19 May 2025.

### 4.2. Bacterial Isolation, Culture Conditions, and Antimicrobial Compounds

For the isolation of *Staphylococcus* spp., samples were cultured on CHROMagar Staph aureus agar (Chebio Fejlesztő Kft., Budapest, Hungary). *Escherichia coli* was isolated using ChromoBio Coliform agar (Biolab Zrt., Budapest, Hungary), whereas *Enterococcus* spp. were isolated on modified m-Enterococcus agar (Merck KGaA, Darmstadt, Germany). Presumptive colonies showing characteristic morphology were subcultured on tryptic soy agar (Biolab Zrt., Budapest, Hungary) to obtain pure cultures. Isolates were preserved in a Microbank system (Pro-Lab Diagnostics, Richmond Hill, ON, Canada) at −80 °C until antimicrobial susceptibility testing. Species identification was performed by matrix-assisted laser desorption/ionization time-of-flight mass spectrometry (MALDI-TOF MS) using a Bruker MALDI Biotyper system and Biotyper software version 12.0 (Bruker Daltonics GmbH, Bremen, Germany), according to the manufacturer’s instructions. Species-level identification was accepted according to the manufacturer’s recommended score thresholds. Where the available MALDI-TOF MS result did not support an unambiguous species-level assignment, the isolate was retained at the highest taxonomic resolution supported by the laboratory record, including genus-level (*Enterococcus* spp.) or species-pair (*E. durans*/*hirae*) assignments.

Antimicrobial reference powders were obtained from Merck KGaA (Darmstadt, Germany). Stock solutions were prepared in accordance with Clinical and Laboratory Standards Institute (CLSI) recommendations for dilution antimicrobial susceptibility testing. Amoxicillin and amoxicillin–clavulanic acid, the latter prepared at a 2:1 ratio, were dissolved in 0.01 mol/L phosphate buffer at pH 7.2, whereas imipenem was dissolved in 0.1 mol/L phosphate buffer at pH 6.0. Ceftriaxone, doxycycline, spectinomycin, neomycin, colistin, tiamulin, tylosin, lincomycin, and vancomycin were dissolved in distilled water.

The potentiated sulfonamide combination was prepared using trimethoprim and sulfamethoxazole at a 1:19 ratio. Sulfamethoxazole was dissolved in hot water supplemented with a few drops of 2.5 mol/L NaOH, whereas trimethoprim was dissolved in distilled water acidified with 0.05 mol/L HCl. Enrofloxacin was dissolved in distilled water containing a few drops of 1 mol/L NaOH. Florfenicol was dissolved using a small amount of 95% ethanol followed by dilution with distilled water. Stock solutions were prepared at 1024 µg/mL after correction for the potency and purity of the individual antimicrobial compounds [35].

The antimicrobial panel comprised amoxicillin, amoxicillin–clavulanic acid, ceftriaxone, imipenem, neomycin, spectinomycin, doxycycline, florfenicol, tylosin, tiamulin, lincomycin, enrofloxacin, colistin, trimethoprim–sulfamethoxazole, and vancomycin.

### 4.3. Broth Microdilution Minimum Inhibitory Concentration Testing and Interpretation

Minimum inhibitory concentrations (MICs) were determined using the broth microdilution method in accordance with CLSI methodology for aerobic bacteria. Frozen isolates were recovered and inoculated into 3 mL of cation-adjusted Mueller–Hinton broth and incubated at 37 °C for 18–24 h before susceptibility testing.

For MIC determination, 90 µL of cation-adjusted Mueller–Hinton broth was dispensed into each well of sterile 96-well microtiter plates, except for the first antimicrobial-containing column, which received 180 µL of broth containing the highest tested antimicrobial concentration. Two-fold serial dilutions were subsequently prepared across the plate through column 10. Column 11 served as the positive growth control, whereas column 12 served as the sterility control.

Bacterial suspensions were adjusted to a turbidity equivalent to a 0.5 McFarland standard and subsequently diluted 1:20 in cation-adjusted Mueller–Hinton broth. Ten microliters of the diluted suspension was inoculated into each 90 µL test well, yielding a final bacterial density of approximately 5 × 10^5^ CFU/mL. Plates were incubated aerobically at 37 °C for 18–24 h. MIC endpoints were determined as the lowest antimicrobial concentration that completely inhibited visible bacterial growth. Plates were evaluated using a SWIN automatic MIC reader and the VIZION system (CheBio Fejlesztő Kft., Budapest, Hungary).

MIC results were interpreted using applicable CLSI clinical breakpoints and European Committee on Antimicrobial Susceptibility Testing (EUCAST) epidemiological cutoff values or interpretive criteria, where available. For antimicrobial–organism combinations for which poultry-specific or species-specific clinical breakpoints were unavailable, interpretation was restricted to combinations supported by applicable clinical or epidemiological criteria. Accordingly, categorical susceptibility results were treated as surveillance descriptors and were not interpreted as direct predictors of therapeutic outcome.

Quality control was performed using *Escherichia coli* ATCC 25922, *Enterococcus faecalis* ATCC 29212, and *Staphylococcus aureus* ATCC 29213. Quality-control strains were included on each testing day, and MIC values were evaluated against the applicable CLSI quality-control ranges. Isolates showing unclear growth endpoints or unexpected results were retested.

MIC distributions were additionally summarized using MIC_50_ and MIC_90_, defined as the lowest antimicrobial concentrations inhibiting 50% and 90% of isolates, respectively. Where validated organism-specific interpretive criteria were available, categorical susceptibility results were used descriptively. Because a complete and internally consistent set of applicable clinical breakpoints or epidemiological cutoff values was not available across all organism–antimicrobial combinations, formal MDR, XDR, and PDR prevalence estimates were not used as primary outcomes. The numeric thresholds recorded in the regional dataset were analyzed separately as an operational sensitivity analysis and are reported exclusively as threshold-positive classifications [28,29].

### 4.4. Whole-Genome Sequencing, Assembly Generation, and Quality Control

Genomic DNA was extracted from bacterial cultures using the Quick-DNA Fungal/Bacterial Miniprep Kit (Zymo Research, Irvine, CA, USA) according to the manufacturer’s instructions. Mechanical disruption was performed using a TissueLyzer LT instrument (Qiagen GmbH, Hilden, Germany) at 50 Hz for 5 min, and purified DNA was stored at −20 °C until library preparation. Sequencing libraries were prepared from 50 ng of genomic DNA using the TruePrep DNA Library Prep Kit V2 for Illumina (TD501-01; Vazyme Biotech, Nanjing, China), with sample indexing performed using the Nextera XT Index Kit v2 Set A and Set B (Illumina, San Diego, CA, USA). Library concentrations were determined fluorometrically using the Qubit dsDNA HS Assay Kit (Thermo Fisher Scientific, Waltham, MA, USA).

Indexed libraries were pooled and converted to MGI-compatible libraries using the MGIEasy Universal Library Conversion Kit (App-A; MGI Tech, Wuhan, China). Following library conversion, circularization and DNA nanoball generation were performed according to the manufacturer’s protocol. DNA nanoball libraries were loaded onto a DNBSEQ-G400RS sequencing flow cell and sequenced on a DNBSEQ-G400 platform (MGI Tech, Wuhan, China) using the HotMPS High Throughput Sequencing Kit FCL PE150, generating 150 bp paired-end reads.

Raw sequencing reads were quality assessed using FastQC v0.11.9 [36] and processed using fastp v0.23.2-3 [37], Bloocoo v1.0.7 [38], and Trim Galore v0.6.6 [39] for quality filtering, error correction, and removal of adapter or low-quality sequences. De novo assembly was performed independently using MEGAHIT v1.2.9 [40] and SPAdes v4.0.0 [41]. The resulting assemblies were subsequently merged using GAM-NGS v1.1b [42] to obtain the final draft assemblies used for downstream analysis. Assembly quality was evaluated using QUAST v5.2 [26] and BUSCO v5 [43], while genome characteristics were additionally assessed using GenomeScope v2.2 [44].

Twenty-four *E. coli* draft assemblies corresponding to chicken-origin isolates from Northern Hungary were included in the present analysis. For the downstream analyses reported here, the merged contig assemblies were used as the genomic input; no additional read-level reprocessing or de novo reassembly was performed. The sequencing and primary assembly workflow had been completed before the present downstream reanalysis. In the project archive accessible for the current study, the final merged draft assemblies and associated downstream analysis outputs had been retained, whereas the original raw-read files had not been retained and were therefore no longer available for reanalysis. Consequently, no new read-level quality control, read mapping, or de novo reassembly could be performed, and sequence-level verification in the present study was restricted to the archived draft assemblies. Each assembly was parsed to verify nucleotide characters and the uniqueness of contig identifiers. Sequences were converted to uppercase and examined for terminal ambiguous nucleotides. Contigs shorter than 200 bp were removed from the analytical assembly set. Retained contigs were renamed using short, isolate-specific identifiers and formatted at 80 nucleotides per line. Original and processed assembly files were checksummed, and an explicit old-to-new contig identifier mapping was retained to preserve isolate- and contig-level traceability.

Assembly quality metrics were recalculated directly from the processed sequences and included total assembly length, number of retained contigs, GC content, length of the largest contig, N50, and L50. FASTA standardization was performed to facilitate downstream sequence analysis and repository preparation; these processing steps were not interpreted as evidence of genome completeness, taxonomic purity, or repository acceptance [27].

Assembly size was additionally examined as a quality-control parameter. Assemblies exceeding 6.5 Mb were flagged for closer inspection but were not truncated solely to conform to an expected *E. coli* genome-size range. Isolate 832 was retained in the genomic dataset despite its markedly fragmented assembly because sequence data were available for this isolate; however, negative gene findings and genome-wide conclusions derived from this assembly were interpreted cautiously. The available Kraken 2/Pavian taxonomic classification reports were reviewed for evidence of possible non-target sequence content [24,25]. Because these reports provided aggregate taxonomic information without sufficiently resolved contig-level assignments for all detected signals, they were not used to remove contigs solely on the basis of taxonomic proportions. Consequently, no assembly was described as formally contamination-free in the absence of a dedicated contig-resolved foreign-sequence screen. The 24 draft genome assemblies were deposited in the National Center for Biotechnology Information (NCBI) under BioProject accession PRJNA1528658.

### 4.5. Resistance-Gene Annotation and Sequence-Context Analysis

Open reading frames were identified using Prodigal v2.6.3 [45]. Antimicrobial resistance-associated determinants were screened using Resistance Gene Identifier (RGI) v5.1.0 against the Comprehensive Antibiotic Resistance Database (CARD) [15] and ABRicate [46], with CARD as the nucleotide reference database. The archived nucleotide-alignment outputs were subsequently reconciled at isolate and contig level using the isolate identifier, original contig identifier, genomic coordinates, and strand orientation. For each reported locus, coordinates were verified against the corresponding assembly sequence and mapped to the standardized contig identifiers generated during FASTA processing.

The resistance-gene analysis retained the reported gene designation, alignment coverage, reference accession, strand orientation, and nucleotide identity where these fields were available in the archived output. Because the retained alignment tables contained the underlying nucleotide-level calls, downstream analyses preserved the reported per-hit coverage and identity values rather than retrospectively assigning new RGI confidence categories. Conserved multidrug-efflux components, global transcriptional regulators, and other intrinsic resistance-associated loci were not automatically interpreted as acquired antimicrobial resistance determinants. CARD was used as the reference framework for resistance-gene annotation [15].

Mobile genetic elements associated with resistance determinants were assessed using MobileElementFinder v1.0.3 [47], and plasmid-origin predictions were generated using PlasFlow v1.1 [16]. MobileElementFinder annotations and PlasFlow classifications were treated as computational predictions and were not interpreted as evidence of a complete transferable element or a closed plasmid.

For selected epidemiologically relevant resistance determinants, the corresponding nucleotide sequences were extracted directly from the assemblies using one-based inclusive genomic coordinates. Sequences encoded on the reverse strand were reverse-complemented before sequence comparison. The five *bla*_CTX-M-1_-labelled intervals identified among the 24 isolates were compared at the nucleotide level and independently checksummed to assess sequence identity.

For isolates containing multiple resistance-associated loci on the same contig, local genetic context was examined using the original contig coordinates together with the available mobile-element annotations. In particular, the resistance-gene region of isolate 1316 was reconstructed at the contig level to assess the spatial relationship among the detected resistance determinants and annotated mobile genetic elements. Gene co-localization was defined conservatively as the presence of two or more determinants on the same assembled contig and was not interpreted as proof of physical linkage on a complete plasmid, horizontal transfer, or clonal transmission. PlasFlow assignments were regarded as sequence-origin predictions rather than definitive plasmid localization, and mobile-element annotations were likewise interpreted as computational predictions rather than independently validated complete mobile genetic elements [16].

### 4.6. Statistical Analysis and Multivariate Visualization

All statistical analyses were conducted at the level of the Northern Hungary regional isolate collection. Because the sampling structure was not designed to support independent farm-level inference, no comparisons among individual farms or holdings were performed.

MIC distributions were analyzed separately for *Staphylococcus* spp., *Enterococcus* spp., and *E. coli*. Pairwise covariation between antimicrobial susceptibility measurements was explored using Spearman rank correlation coefficients. Correlations were calculated from the recorded MIC values rather than from dichotomized susceptibility categories. Antimicrobial variables without sufficient variation for calculation of a meaningful correlation coefficient were excluded from the respective pairwise comparison. Correlation coefficients were interpreted descriptively and were not considered evidence of genetic linkage, co-selection, or a shared mobile genetic element.

The multidimensional structure of the *E. coli* susceptibility profiles was further explored by principal component analysis. The ten antimicrobial agents constituting the predefined *E. coli* analytical panel were included. MIC values were log2-transformed and subsequently centered and scaled to unit variance before dimensionality reduction. Principal components were calculated by singular-value decomposition. Whole-genome sequencing availability was visualized as isolate-level metadata in the ordination plots but was not used to define clusters, supervise the dimensionality-reduction procedure, or train a predictive classifier.

The composition of the assembly-linked and non-sequenced *E. coli* subsets was summarized descriptively according to specimen type and sampling year. These comparisons were used to characterize the available genomic subset and were not interpreted as demonstrating statistical representativeness of the 24 sequenced isolates for the complete collection of 69 *E. coli*. Similarly, because the available metadata did not support a robust hierarchical model accounting for within-farm or within-flock dependence, no inferential tests were used to estimate regional prevalence or farm-level effects from isolate-level observations.

Phenotype–genotype relationships were evaluated primarily at the isolate level by comparing recorded MIC profiles with the presence of selected resistance-associated determinants. Formal estimates of diagnostic sensitivity, specificity, positive or negative predictive value, or genotype–phenotype concordance were not calculated when a reliable binary genomic reference standard could not be established. In particular, absence of a gene call was not considered definitive evidence of gene absence in assemblies of limited completeness or continuity.

## 5. Conclusions

Chicken-associated commensal bacteria sampled in Northern Hungary displayed heterogeneous MIC distributions, with a particularly marked separation between the ceftriaxone MIC_50_ (0.031 µg/mL) and MIC_90_ (512 µg/mL) in *E. coli*. This contrast demonstrates that reliance on MIC_50_ alone would obscure a smaller high-MIC subpopulation, including isolates carrying *bla*_CTX-M-1_-labelled intervals, and therefore underscores the value of preserving complete MIC distributions rather than relying on a single summary statistic in antimicrobial-resistance surveillance. The available assembly subset added positive sequence evidence: five *bla*_CTX-M-1_-labelled intervals were identical over 876 bp, and a single contig in isolate 1316 carried seven reported resistance-gene intervals, including a TEM-family locus within a Tn2 annotation. These observations support integrated, isolate-linked susceptibility and sequence surveillance. They do not establish the population prevalence of resistance across the region, complete plasmid structures, or direct transmission. Retaining uncertain material, including isolate 832, is compatible with rigorous reporting only when the strength of positive observations is separated from the limitations of negative genomic inference.

## Figures and Tables

**Table 1:** Composition of the Northern Hungary chicken collection.

Bacterial Group	Isolates	Tracheal	Cloacal	2022	2023	Assemblies
*Staphylococcus* spp.	26	26	0	26	0	0
*Enterococcus* spp.	71	35	36	43	28	0
*Escherichia coli*	69	23	46	49	20	24
Total	166	84	82	118	48	24

All records were assigned to adult egg-producing chickens. Isolate counts are not independent farm or animal denominators.

**Table 2:** Recorded MIC_50_/MIC_90_ values (µg/mL). MIC–minimum inhibitory concentration.

Antimicrobial	*Staphylococcus* (n = 26)	*Enterococcus* (n = 71)	*Escherichia coli* (n = 69)
Amoxicillin	0.063/0.125	0.5/2	64/512
Amoxicillin–clavulanic acid	0.031/0.25	0.5/2	8/128
Ceftriaxone	2/16	32/1024	0.031/512
Imipenem	0.063/0.25	1/4	0.25/1
Neomycin	1/16	128/1024	64/128
Spectinomycin	128/256	64/256	64/512
Doxycycline	0.016/4	8/32	4/256
Florfenicol	2/4	4/32	16/512
Tylosin	1/8	2/256	512/1024
Tiamulin	64/128	128/512	256/1024
Lincomycin	32/512	32/512	1024/1024
Enrofloxacin	0.125/16	2/32	0.031/2
Colistin	32/512	128/1024	0.25/1024
Trimethoprim–sulfamethoxazole	4/16	16/1024	4/1024
Vancomycin	0.25/1	1/4	256/512

Values are observed order statistics, not interpolated estimates. The entry 1024 is preserved as recorded because its off-scale meaning could not be independently established. High values for intrinsically unsuitable organism–agent combinations are not evidence of newly acquired resistance.

**Table 3:** *bla*_CTX-M-1_-labelled intervals and linked ceftriaxone minimum inhibitory concentration (MIC) records.

Isolate	Ceftriaxone MIC	Prepared Contig	Original Interval (bp; Strand)	Length (bp)	Coverage/ Identity (%)
1306	512	EC1306_ctg00169	1429–2304 (−)	876	100/100
1308	512	EC1308_ctg01473	929–1804 (−)	876	100/100
1310	256	EC1310_ctg00112	34,166–35,041 (−)	876	100/100
1312	1024	EC1312_ctg01470	1794–2669 (+)	876	100/100
1315	32	EC1315_ctg00438	2320–3195 (+)	876	100/100

Coordinates are one-based and inclusive and are unchanged within the retained contigs. All five strand-corrected intervals were identical. Coverage and identity are the values in the alignment output, not results of a new database search. Prepared contig names map to original names in Supplementary File S1.

**Table 4:** Reported resistance-gene intervals on the 1316 contig EC1316_ctg00050.

Reported Xall	Original Coordinates (bp)	Strand	Coverage (%)	Identity (%)
*sul2*	54,504–55,319	+	100	99.63
APH(3″)-Ib	55,380–56,183	+	100	99.75
APH(6)-Id	56,183–57,019	+	100	99.88
*dfrA1*	58,266–58,739	+	100	100
ANT(3″)-IIa	58,822–59,623	+	82.51	99.75
TEM-family	60,084–60,944	−	100	99.88
*tet(A)*	66,190–67,436	+	97.8	100

Original contig: PairedContig_51. The first-to-last gene span was 12,933 bp. Computational annotations placed IS26 at 51,988–52,807 and Tn2 at 59,937–64,886. These are sequence annotations, not independently validated complete mobile elements.

## Data Availability

The whole-genome assembly data of the 24 *Escherichia coli* isolates analyzed in this study have been deposited in the National Center for Biotechnology Information (NCBI) under BioProject accession PRJNA1528658. The isolate-level phenotypic MIC data, genomic analysis outputs, and supporting datasets underlying the findings of this study are provided in the Supplementary File S1.

## Supplementary Materials

The following supporting information can be downloaded at: https://www.mdpi.com/article/10.3390/antibiotics15101002/s1. Supplementary File S1: Supporting datasets and analysis outputs underlying the phenotypic and genomic analyses (Excel workbook; 17 worksheets). Figure S1: Spearman rank correlation matrix of recorded MICs in *Staphylococcus* spp. Figure S2: Spearman rank correlation matrix of recorded MICs in *Enterococcus* spp. Figure S3: Isolate-level heatmap of recorded MIC profiles for the 69 *Escherichia coli* isolates, with assembly availability indicated. Figure S4: Specimen-type composition of the assembly-linked and non-sequenced *Escherichia coli* subsets.

## Abbreviations

The following abbreviations are used in this manuscript.

AMR	Antimicrobial Resistance
CARD	Comprehensive Antibiotic Resistance Database
ESBL	Extended-spectrum β-lactamase
FASTA	Sequence text-file format
MIC	Minimum inhibitory concentration
MDR	Multidrug resistance
N50	Contig length at which half of the assembly is contained in contigs of that length or longer
PCA	Principal component analysis
QC	Quality control
